# Crystal structure and induced stability of trimeric BxpB: implications for the assembly of BxpB-BclA complexes in the exosporium of *Bacillus anthracis*

**DOI:** 10.1128/mbio.01172-23

**Published:** 2023-06-29

**Authors:** Debasish Chattopadhyay, Dionna R. Walker, Shane T. Rich-New, John F. Kearney, Charles L. Turnbough, Jr.

**Affiliations:** 1 Department of Medicine, University of Alabama at Birmingham, Birmingham, Alabama, USA; 2 Department of Microbiology, University of Alabama at Birmingham, Birmingham, Alabama, USA; Pennsylvania State University, University Park, Pennsylvania, USA

**Keywords:** *Bacillus anthracis*, exosporium, BxpB, crystal structure, BclA

## Abstract

The outermost exosporium layer of *Bacillus anthracis* spores, the causative agents of anthrax, is comprised of a basal layer and an external hair-like nap. The nap includes filaments composed of trimers of the collagen-like glycoprotein BclA. Essentially all BclA trimers are attached to the spore in a process in which part of the 38-residue amino-terminal domain (NTD) of BclA forms an extremely stable interaction with the basal layer protein BxpB. Evidence indicates that the BclA-BxpB interaction is direct and requires trimeric BxpB. To further investigate the nature of the BclA-BxpB interaction, we determined the crystal structure of BxpB. The structure was trimeric with each monomer consisting of 11 β strands with connecting loops. The structure did not include apparently disordered amino acids 1–19, which contain the only two cysteine residues of the 167-residue BxpB. The orientation of the structure reveals regions of BxpB that could be involved in interacting with the BclA NTD and with adjacent cysteine-rich proteins in the basal layer. Furthermore, the BxpB structure closely resembles that of the 134-residue carboxyl-terminal domain of BclA, which forms trimers that are highly resistant to heat and detergent. We demonstrated that BxpB trimers do not share this resistance. However, when BxpB trimers are mixed with a peptide containing residues 20–38 of BclA, they form a complex that is as stable as BclA-BxpB complexes extracted from spores. Together, our results provide new insights into the mechanism of BclA-BxpB attachment and incorporation into the exosporium.

**IMPORTANCE:**

The *B. anthracis* exosporium plays major roles in spore survival and infectivity, but the complex mechanism of its assembly is poorly understood. Key steps in this process are the stable attachment of collagen-like BclA filaments to the major basal layer structural protein BxpB and the insertion of BxpB into an underlying basal layer scaffold. The goal of this study is to further elucidate these interactions thereby advancing our understanding of exosporium assembly, a process shared by many spore-forming bacteria including important human pathogens.

## INTRODUCTION

The Gram-positive, aerobic soil bacterium *Bacillus anthracis* forms spores when starved for nutrients and contact with these spores can cause the potentially lethal disease anthrax in animals and humans. Sporulation starts with an asymmetric septation that divides the vegetative cell into two genome-containing compartments called the mother cell and forespore. The mother cell then engulfs the smaller forespore and surrounds it with three protective layers: a peptidoglycan-containing cortex, a closely apposed proteinaceous coat, and a loosely fitting exosporium (1). Subsequent lysis of the mother cell releases a dormant spore capable of surviving under harsh conditions for many years. Upon encountering a nutrient-rich aqueous environment, spores can rapidly germinate and grow as vegetative cells.

The outermost exosporium layer of *B. anthracis* spores plays key roles in spore viability (2, 3) and apparently in the progression of disease within an infected host (4, 5). It also serves as the source of molecular markers used to detect *B. anthracis* spores (6, 7), a preferred weapon of bioterrorism and biological warfare. The exosporium is a prominent bipartite structure comprised of a paracrystalline basal layer and an external hair-like nap (8). Each filament of the nap is formed solely by a trimer of the collagen-like glycoprotein BclA (9 - 11). BclA is composed of three domains: a 38-residue amino-terminal domain (NTD), a central collagen-like region containing a strain-specific number of triplet amino-acid repeats, and a 134-residue carboxy-terminal domain (CTD) that promotes trimer formation (10, 12, 13). The collagen-like region and CTD are glycosylated (14, 15). In contrast to the nap, the basal layer of the exosporium contains approximately 25 different proteins (16). One of these proteins is BxpB (also called ExsFA), which is required for the attachment of approximately 98% of the BclA present in the exosporium (17, 18). In this process, each filament of the nap is attached to a basal layer surface protrusion that appears to be formed by a trimer of BxpB (9).

Basal layer attachment of BclA occurs through, and requires only, its NTD (12, 19, 20). Efficient attachment requires proteolytic cleavage between BclA residues 19 and 20 (19), which occurs only after the NTD is bound to the developing forespore (20). In mature wild-type spores, BclA is included in high molecular mass (>250-kDa) complexes that also include BxpB and in some cases other exosporium proteins, such as ExsY and its homolog CotY (17, 21). These complexes are resistant to heat, detergents, and reducing agents, conditions designed to dissociate non-covalently bound protein complexes and to reduce disulfide bonds. These results suggested that BclA and BxpB are attached through a non-disulfide covalent bond (16, 17), although attempts to identify such a bond have been unsuccessful.

To further investigate the nature of the BclA-BxpB attachment, we determined the crystal structure of BxpB. The resulting trimeric structure revealed surfaces that could physically interact with the NTD of BclA and with other basal layer proteins. The structure of the BxpB monomer closely resembles that of the BclA CTD, which forms extremely stable trimers (12). BxpB trimers do not share this stability; however, when mixed with a segment of the BclA NTD, they form complexes as stable as BclA-BxpA complexes found in spores. We discuss all these results in terms of mechanisms of BclA-BxpB complex formation and insertion into the exosporium.

## RESULTS

### Crystal structure of a BxpB trimer

**Fig 1:**
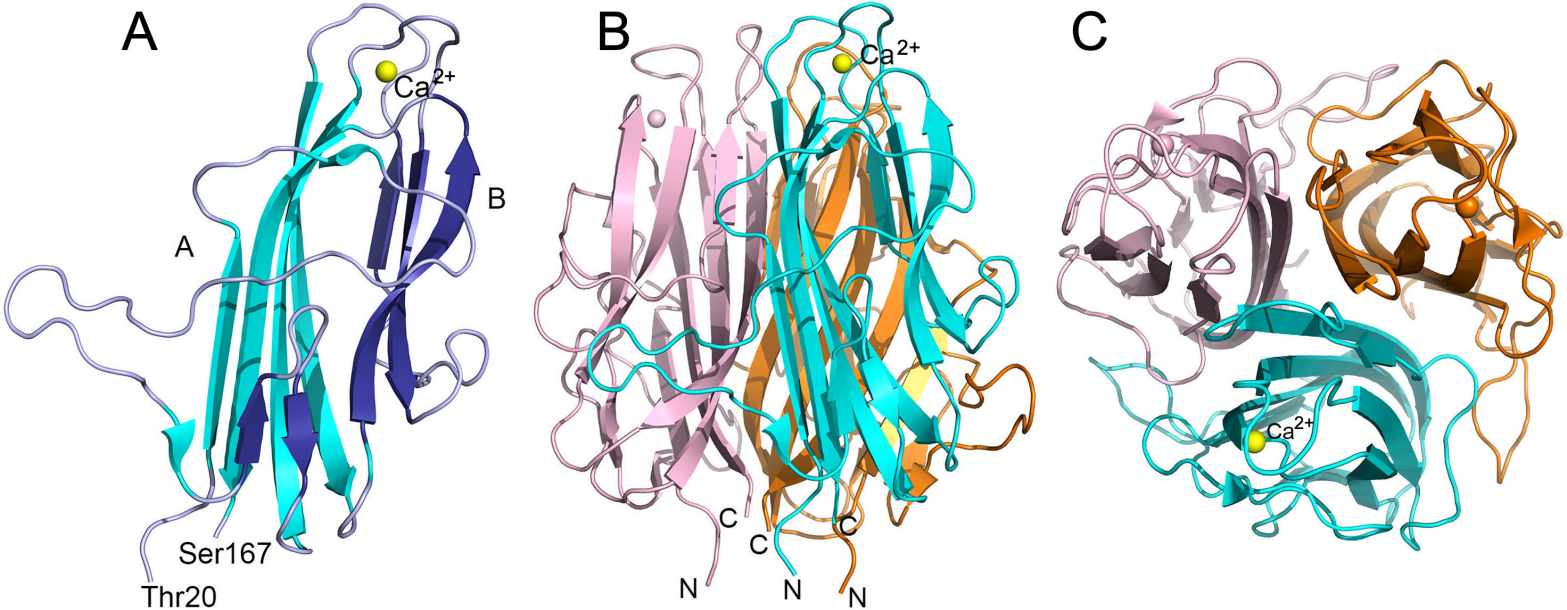
Crystal structures of BxpB. (**A**) Diagram showing the structure of the BxpB monomer. N-terminal 19 residues could not be modeled presumably because of disorder. BxpB residues 20 to 167 fold into a jelly roll-like structure consisting of two antiparallel β sheets, A and B, composed of six (cyan) and five (dark blue) β strands, respectively. One Ca^2+^ ion (yellow sphere) is hexa-coordinated by amino acids located on two β turns connecting the β sheets. β sheet B exhibits two β hairpins (residues 61–63, 68–70 and 95–102, 114–117) as indicated by analysis of the structure using PROMOTIF (22). The N- and C-terminal residues of BxpB are labeled. In the crystal structure, residue 20 was modeled as Ala (instead of Thr) as there was no density for the sidechain. Also see topology diagram in Fig. S1. (**B**) Diagram showing the BxpB trimer formed by threefold symmetry related BxpB monomers in the crystal structure. Monomers are colored cyan, orange, and light pink. N and C termini are labeled. The Ca^2+^ ion of each monomer is shown as a sphere, with the yellow sphere labeled. (**C**) Diagram of the BxpB trimer rotated forward 90° relative to panel B to provide a top view of the structure.

An amino-terminally His_6_-tagged version of the BxpB protein of *B. anthracis* was expressed in *Escherichia coli*, affinity purified, and cleaved with Factor Xa protease to precisely remove the His_6_ tag and adjacent Xa cleavage site. The His_6_ tag and Factor Xa were removed by affinity capture. The resulting highly purified 167-residue BxpB, in Factor Xa cleavage buffer (20 mM Tris-HCl, pH 8.0, 100 mM NaCl, 2 mM CaCl_2_, hereafter Xa buffer) containing 20 mM dithiothreitol (DTT), was used for crystallization. X-ray diffraction data were collected from a single frozen crystal (see Materials and Methods and Table S1). The structure of BxpB was solved by molecular replacement using a homology model and refined to 1.4 Å resolution (PDB ID: 8D02). Although it was confirmed by mass spectrometry that the protein used for crystallization contained all 167 residues (data not shown), the structure included only residues 20–167. The crystal structure displayed a trimeric assembly of BxpB monomers (Fig. 1A and B). Each monomer folds in a jelly roll-like structure composed of two antiparallel β sheets, labeled A and B, containing six and five strands, respectively (Fig. 1A; Fig. S1). β sheet B includes two β hairpin motifs. A novel feature of the BxpB crystal structure is a bound calcium ion hexa-coordinated by oxygen atoms of residues Asp87, Ser89, Glu94, and Thr153 located on neighboring β turns (Fig. S2). The side view in Fig. 1A shows that the BxpB termini represented by residues Thr20 and Ser167 are close together at the bottom of the image. The top view of the trimeric structure shows that the β sheets of each monomer form a tight core within the trimeric structure (Fig. 1C).

### BxpB is a trimer in solution

**Fig 2:**
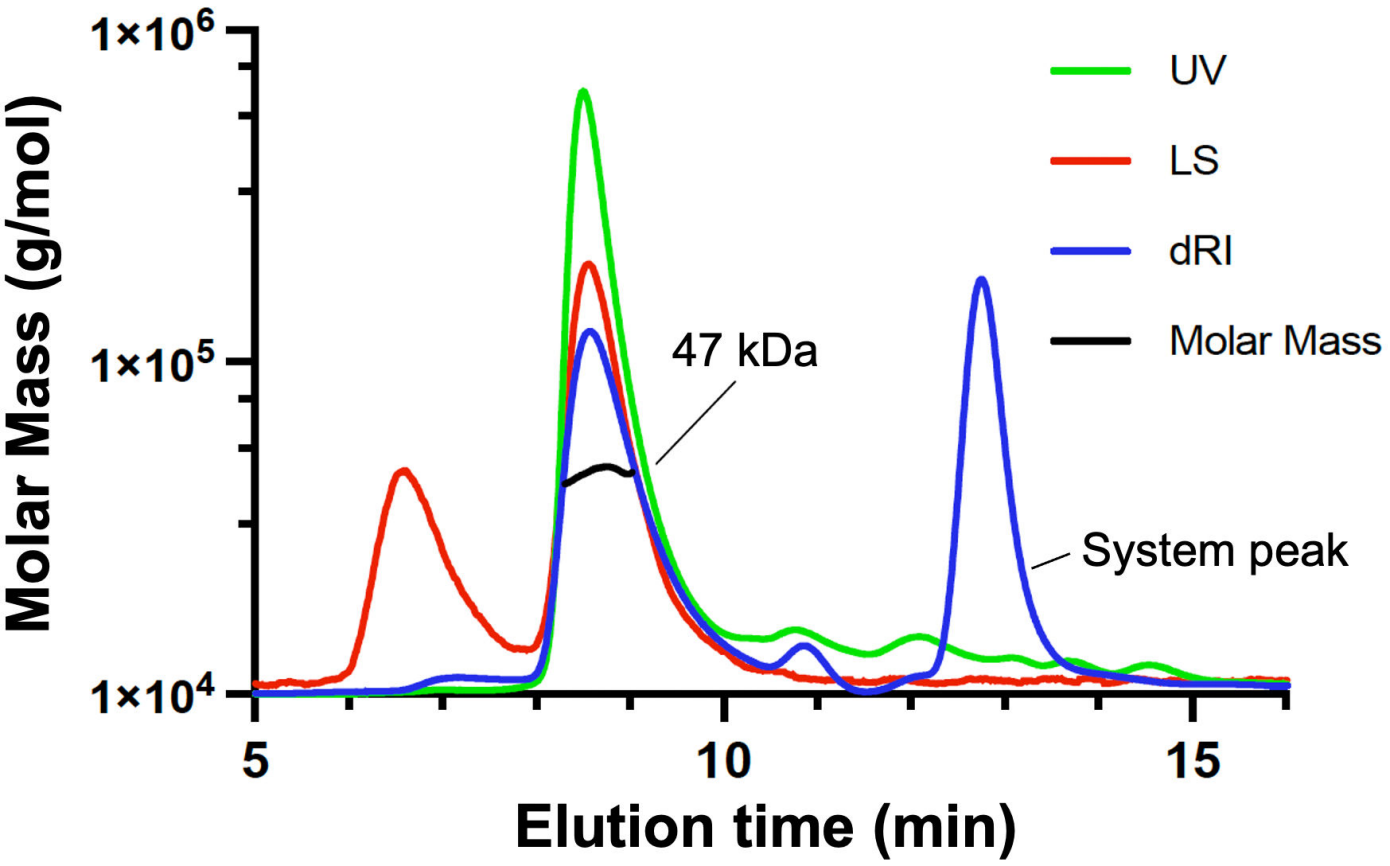
SEC-MALS analysis of BxpB in Xa buffer. BxpB was separated (eluted with Xa buffer containing 2 mM TCEP) and analyzed using an analytical SEC column. The chromatogram displays UV at 280 nm (green), light scattering (red), differential refractive index (blue), and calculated molar mass (black). Nearly all BxpB (i.e., UV-absorbing material) elutes at 8.5 min with a calculated mass of 47 kDa ± 2, close to that expected for a BxpB trimer. The artifactual system peak is labeled.

To test if the trimeric assembly of BxpB (17.3 kDa) in the crystal structure is an artifact of crystallization, we confirmed the oligomeric state of BxpB in solution. A sample of BxpB (0.26 mg/mL) in Xa buffer containing 2 mM Tris(2-carboxyethyl)phosphine (TCEP) was analyzed by size exclusion chromatography with multi-angle light scattering (SEC-MALS). This technique uses UV absorbance, differential refractive index, and multi-angle light scattering to provide an absolute measurement of the molecular mass of a protein or protein complex (23). The results indicate that essentially all protein in the sample (as judged by the UV scan) migrates as a single species with a molecular mass of 47 kDa ± 2% (Fig. 2). This mass is nearly the same as that expected for a BxpB trimer, which is 52 kDa, indicating that the trimer is a stable and preferred oligomeric state of BxpB. The only other protein species exhibiting a strong light scattering signal appeared to be large aggregates of BxpB with a molecular mass of 1.8 × 10^6^ Da. Judged by the UV scan, the amount of protein in this peak was extremely small. The UV scan also indicated the presence of a small amount of protein smaller (slower eluting) than the BxpB trimer; however, the molecular mass of this material could not be determined. Finally, the large differential refractive index peak near the end of the chromatogram is not due to protein but to a system peak resulting from compressed gas in the sample or small differences between sample and system buffers.

We also used SEC-MALS to examine a sample of BxpB (0.48 mg/mL) in a different buffer, namely, PBS containing 2 mM TCEP. BxpB trimers were again detected, although they appeared somewhat less stable than those formed in Xa buffer (Fig. S3). The calculated molecular mass for BxpB trimers in PBS was 49 kDa ± 2%, slightly closer to the predicted mass than that observed in Xa buffer.

### Comparing structures of BxpB and the BclA CTD

A previously reported I-TASSER prediction of monomeric BxpB structure indicated that it was homologous to the determined crystal structure of the CTD of BclA (9). The 134-residue BclA CTD folds into an all-β structure with a jelly roll topology (24). The structure includes 13 β strands arranged in three antiparallel β sheets. The BclA CTD crystallizes as a tight, globular trimer with the buried core formed by the β strands from each monomeric unit. The N and C termini come together on the side of the trimer that faces the basal layer (12). No cations (e.g., calcium) were found bound to the protein in the crystal structure.

**Fig 3:**
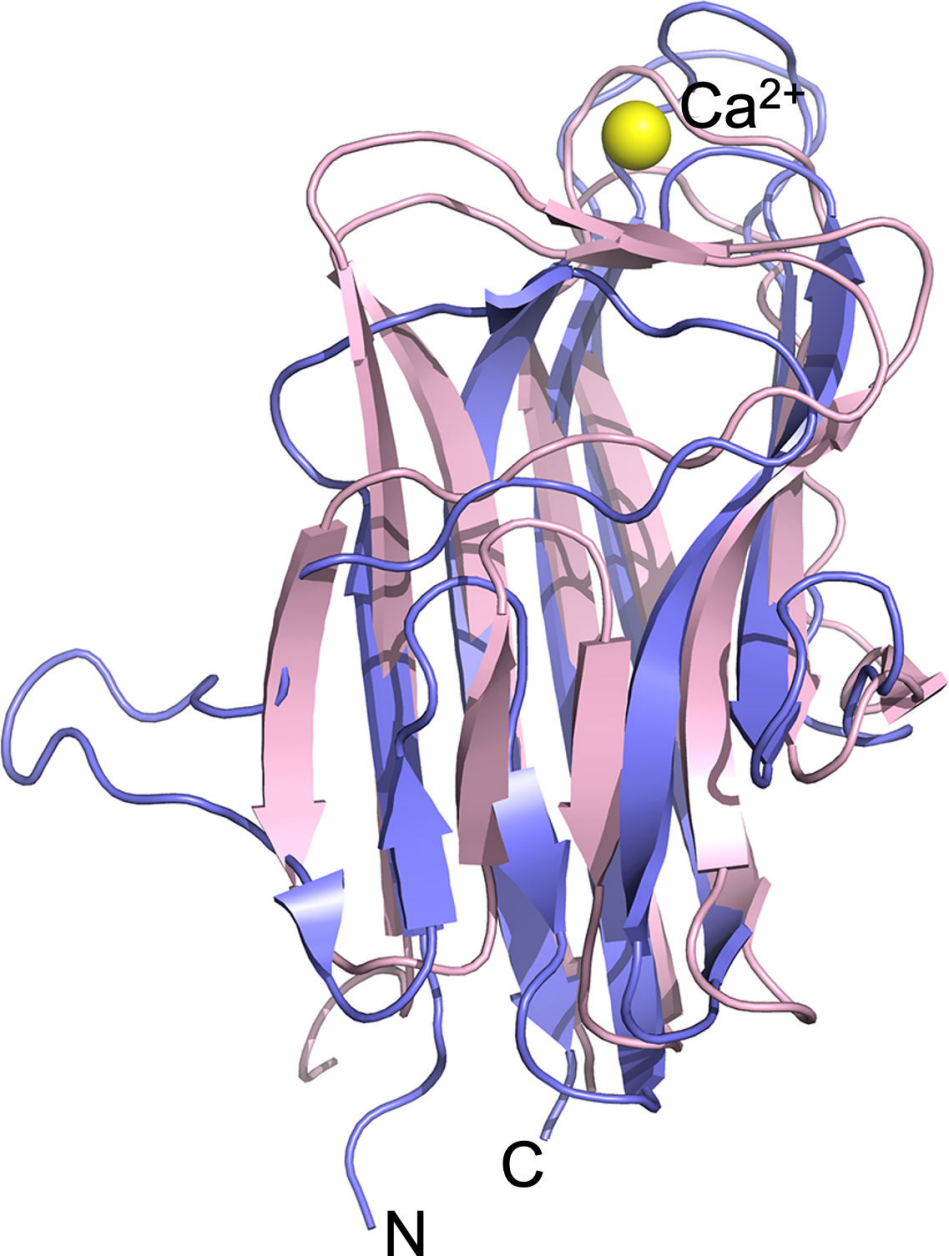
Overlay of BxpB (purple) and BclA CTD (pink) monomeric crystal structures. The Ca^2+^ ion bound to BxpB (yellow sphere) is shown, and the N and C termini of each protein are at the bottom of the image.

The results of the current study now allow a comparison of experimentally determined crystal structures of BxpB and the BclA CTD. An overlay of monomeric structures of BxpB and the BclA CTD showed that overall structures including the orientation of β strands, loops, and the termini are similar (Fig. 3). For both proteins, the β sheets of each monomer pack to form the trimer (as in Fig. 1C). However, the root-mean-square deviation measured for the two monomeric structures is 6.84, indicating significant variations in local structures.

### Comparing stabilities of trimers of BxpB and the CTD of BclA

**Fig 4:**
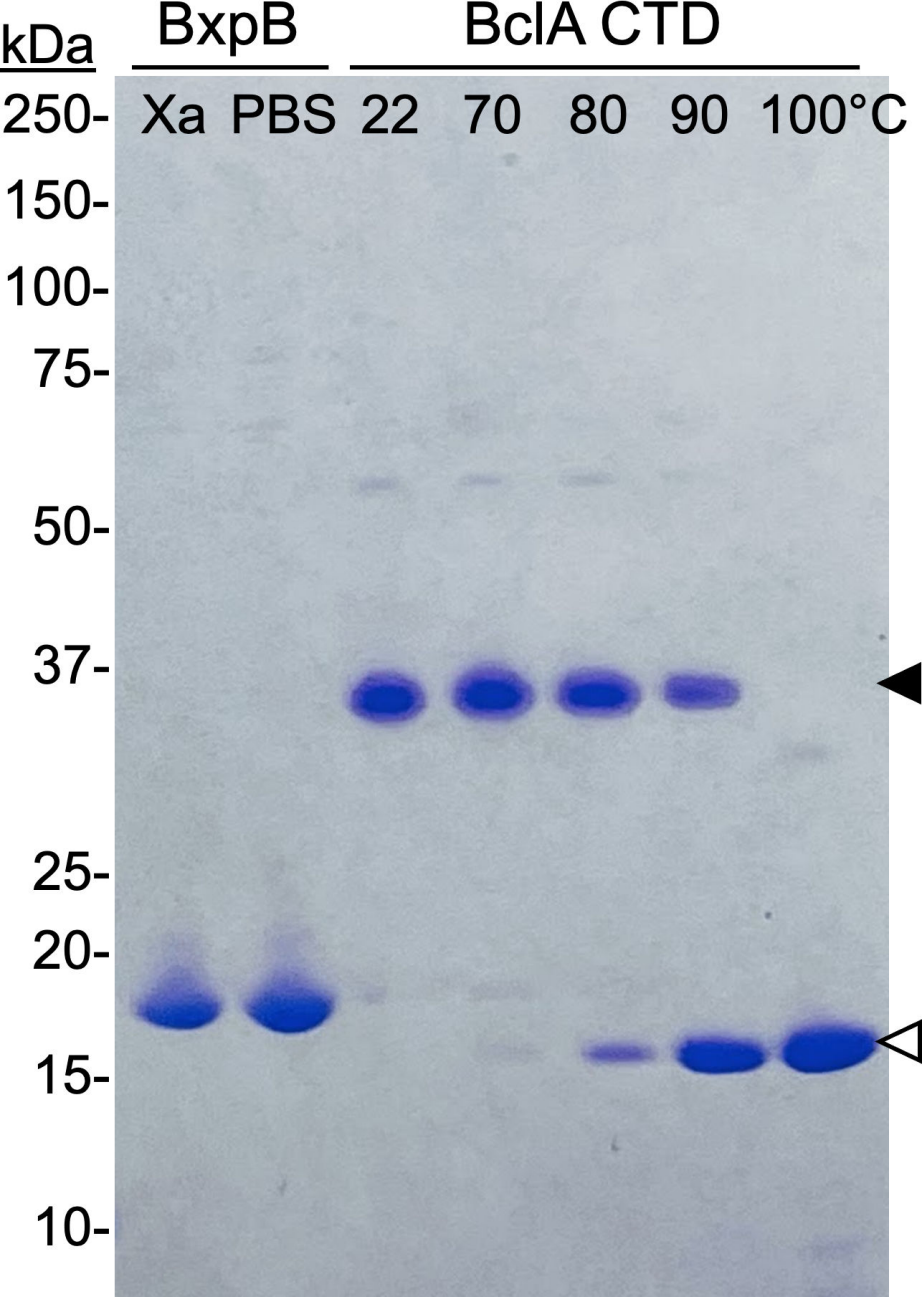
Comparing the stabilities of BxpB trimers and trimers of the BclA CTD. Samples of BxpB trimers in either Xa buffer or PBS and BclA CTD trimers in Xa buffer were made 1× in sample buffer. Portions of the BclA CTD trimer sample were heated at the indicated temperatures for 8 min. A fraction of each sample containing approximately the same amount of protein was analyzed by SDS-PAGE; the Coomassie-stained gel is shown. Filled and open arrowheads indicate the positions of trimeric and monomeric BclA, respectively. Trimers of the BclA CTD migrate faster than predicted from their mass, as previously described (12). The molecular masses of protein standards are indicated.

A hallmark of trimers formed by the CTD of BclA is extreme stability. When heated for 8 min in PBS or in sample buffer containing 2% sodium dodecyl sulfate (SDS) and then analyzed by SDS-PAGE, the *T*_*m*_ values for CTD trimers were 95°C and 84°C, respectively (12). In sharp contrast, when BxpB trimers (2 mg/mL) in either Xa buffer or PBS were mixed with 0.25 vol of 5X sample buffer (final SDS and BxpB concentrations of 2% and 1.6 mg/mL, respectively) and immediately analyzed by SDS-PAGE without heating, virtually all BxpB ran as a 17-kDa monomer (Fig. 4). For direct comparison, a sample of BclA CTD trimers (2 mg/mL) in Xa buffer was mixed with 0.25 vol of 5X sample buffer, portions were heated from 22 to 100°C for 8 min, and samples were cooled to room temperature and immediately analyzed by SDS-PAGE (Fig. 4). The results show a *T*_*m*_ of approximately 85°C, like that previously reported for BclA trimers examined under similar conditions. Overall, under the conditions examined, BxpB trimers are much less stable than trimers of the BclA CTD.

### BxpB trimers and a peptide containing residues 20–38 of the BclA NTD form stable complexes *in vitro*

**Fig 5:**
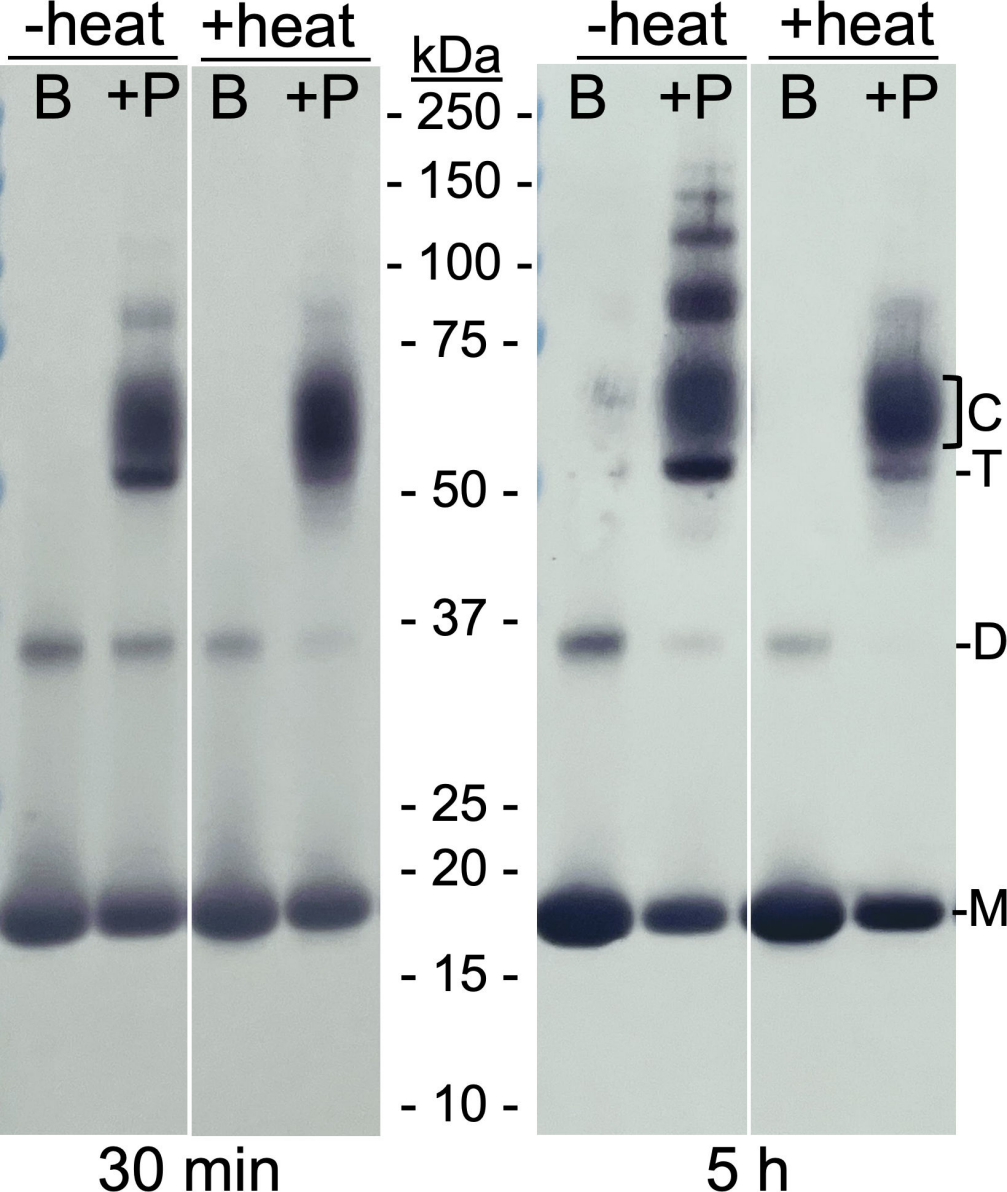
Formation of stable complexes between BxpB trimers and a peptide containing BclA NTD residues 20–38 *in vitro*. A reaction mixture containing trimeric BxpB and a 5× molar excess of a peptide containing BclA residues 20–38 was incubated at room temperature for the indicated time (i.e., 30 min and 5 h), made 1× in sample buffer, and a portion heated as described in the text. Same-sized portions of each unheated and heated mixture of BxpB and peptide (+P) and of unheated and heated BxpB alone (**B**) were analyzed by western blotting with an anti-BxpB mAb. The molecular masses of protein standards are indicated. The positions of BxpB monomers (**M**), dimers (**D**), and trimers (**T**) and of the heat-stable BxpB-peptide complexes (**C**) are also marked.

The NTD of BclA, specifically residues 20–38, forms an extremely stable complex with BxpB during sporulation of *B. anthracis* (12, 19, 20). To determine if this complex could be recapitulated *in vitro*, we mixed equal volumes of a 0.2 mM BxpB solution (in Xa buffer) and a 1 mM solution of a peptide containing BclA residues 20–38 (in water) and incubated the sample at room temperature (22°C). After 30 min and 5 h of incubation, a portion of the reaction mixture was removed and mixed with 0.25 vol of 5X sample buffer. For each time point, this sample was divided into two aliquots with one heated at 100°C for 8 min. As a control, we prepared a 0.1 mM solution of BxpB (in 0.5X Xa buffer), which was mixed with sample buffer as above and divided into two aliquots, with one heated at 100°C for 8 min. Equal portions of all samples were analyzed by SDS-PAGE and western blotting with an anti-BxpB mAb (Fig. 5).

The results show that in the unheated samples containing the BclA peptide, a large fraction of BxpB is present in apparently multimeric forms. These include a distinct band with an apparent molecular mass of 52 kDa, the mass expected for a BxpB trimer. Immediately above the putative trimer band, a wide and pronounced band, with a molecular mass from approximately 55 to 65 kDa, was detected. A gel slice containing this material was excised from the gel, treated *in situ* with chymotrypsin, and analyzed by liquid chromatography with tandem mass spectrometry (LC-MS/MS), which showed the presence of the BclA NTD peptide. Most likely, the 55 to 65-kDa band represents a complex between BxpB trimers and one or more (probably three) molecules of the BclA peptide. These complexes could be the source of the putative BxpB trimers. Additionally, bands of BxpB-containing material were detected with apparent molecular masses above 75 kDa. These bands were much more abundant in the 5 h sample, and their diffuse shapes and apparent masses suggest that they are larger aggregates of BxpB and the BclA peptide. Based on their apparent masses, some of these larger aggregates could be multimers of the putative BxpB trimer-BclA peptide complex.

Inspection of the heated samples showed that the effect of heat (in the presence of detergent) on the various forms of multimeric BxpB was different. The putative (52 kDa) BxpB trimers and >75-kDa species were almost completely destabilized. A similar effect was seen on dimeric BxpB, which can be seen in both the BxpB only and BxpB plus BclA peptide lanes. In contrast, the putative (55 to 65-kDa) BxpB trimer-BclA peptide complexes appeared to be unaffected by the heat treatment. This stability mimics that of BxpB-BclA complexes isolated from *B. anthracis* spores (17). A gel slice containing the heat-treated 55 to 65-kDa band was also analyzed by LC-MS/MS as above, which confirmed the presence of the BclA NTD peptide and identified peptides that covered the entire BxpB amino acid sequence.

### BxpB trimers and a peptide containing residues 20–38 of the BclA NTD form stable complexes *in vivo*

**Fig 6:**
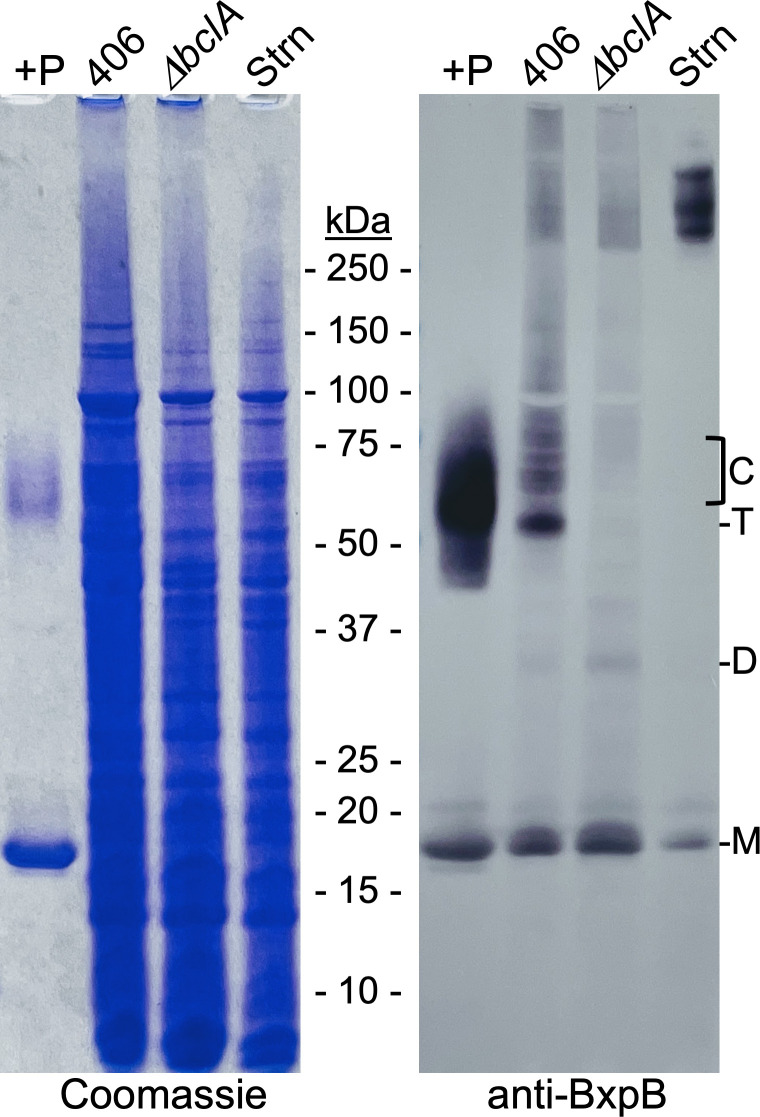
Formation of BxpB trimers and BxpB trimer-BclA NTD peptide complexes *in vivo*. A heat-treated reaction mixture containing BxpB plus a peptide containing BclA residues 20–38 (+P) and exosporium proteins extracted from an equal number of spores of the CLT406, CLT306 (Δ*bclA*), and wild-type Sterne (Strn) strains of *B. anthracis* were analyzed by SDS-PAGE and western blotting with an anti-BxpB mAb (see text for additional details). Both the Coomassie-stained gel and a western blot are shown. The molecular masses of protein standards are indicated. The positions of BxpB monomers (**M**), dimers (**D**), and putative trimers (**T**) and of the apparent heat-stable BxpB-BclA NTD peptide complexes (**C**) are also marked.

To demonstrate that the same stable BxpB-BclA peptide complexes formed *in vitro* could be formed in sporulating cells, we constructed a mutant version of the *B. anthracis* Sterne strain (designated CLT406) in which a stop codon was introduced after codon 38 of the chromosomal *bclA* gene. Instead of full-length BclA, this strain produces a peptide containing BclA residues 1–38, which should be normally cleaved between residues 19 and 20 during spore formation. Purified spores of this strain were produced and mixed with 1× sample buffer, and exosporium proteins were extracted by heating at 100°C for 8 min. As controls, we extracted exosporium proteins from spores of the Sterne stain and from a *ΔbclA* variant unable to produce BclA. In addition, we prepared more heat-treated BxpB-BclA peptide complexes *in vitro* as described above (except incubation was for 1 h). All samples were analyzed by SDS-PAGE and western blotting with an anti-BxpB mAb (Fig. 6). This mAb does not detect the BxpB paralog ExsFB under the conditions employed (Fig. S4).

The western blot showed striking similarities between the bands from the CLT406 and *in vitro* samples. Most importantly, the CLT406 lane contained a series of distinct bands with the same apparent aggregate mass as that of the wide band of putative BxpB trimer-BclA peptide complexes formed *in vitro*. The CLT406 sample also contained a major band at approximately 52 kDa that was presumed to be BxpB trimers. This band was as pronounced as the BclA trimer band detected in an unheated *in vitro* reaction mixture (Fig. 5). Distinct bands of the putative BxpB trimer-BclA peptide complexes and BxpB trimer were not detected in the *ΔbclA* and Sterne samples, indicating the dependence of these species on the BclA NTD peptide. Other BxpB-containing material, including monomeric and dimeric BxpB and uncharacterized species larger than BxpB trimer-BclA peptide complexes, was present in similar amounts in the CLT406 and *ΔbclA* samples. In the case of Sterne spores, except for a small amount of monomer, BxpB was restricted to >250-kDa complexes previously shown to contain BxpB and BclA (17). Taken together, the results above confirm that similar heat-stable BxpB-BclA NTD peptide complexes are formed *in vitro* with purified components and in sporulating cells.

## DISCUSSION

The crystal structure of BxpB provides important new insight into its functioning in exosporium formation. The structure provided the first direct evidence that BxpB forms trimers, which we confirmed as a preferred oligomeric state in solution by SEC-MALS analysis. A trimeric structure for BxpB implies a one-to-one correspondence with attached trimeric BclA filaments. A trimeric structure for BxpB was also predicted from cryo-electron microscopic analyses of exosporia from *B. anthracis* and closely related *Bacillus* species (9, 25, 26). Analysis of exosporia from wild-type and mutant (*ΔbclA* and *ΔbxpB*) *B. anthracis* spores identified the aforementioned basal layer protrusions to which BclA filaments are attached. Evidence that these protrusions were BxpB trimers was that they were present in wild-type and *ΔbclA* exosporia but not in the *ΔbxpB* exosporium and their volume was equal to that calculated for a BxpB trimer (9). BxpB trimers were also proposed to occupy positions of threefold symmetry within a two-dimensional array of hexagonal subunits that comprise the predominant scaffold of the basal layer (9, 25, 26). The hexagonal subunits of this scaffold appear to be formed by self-assembly of the cysteine-rich protein ExsY and stabilized by disulfide bonding (26). The positioning of BxpB trimers within the basal layer appears strategic in that, in the absence of BxpB, the major basal layer scaffold is disordered (9) and multiple exosporium proteins are aberrantly localized (16, 27, 28). It was proposed that BxpB trimers act as a glue that links ExsY hexameric rings together allowing proper assembly of other exosporium proteins (9, 26).

With respect to the role of BxpB trimers in the assembly of the exosporium overall, it should be noted that the ExsY/BxpB scaffold described above appears to be the major structural element of most but not all of the developing exosporium. The exosporium is divided into two distinct domains: a cap, which is comprised of the first approximately 25% of the exosporium formed and covers one end of the forespore, and a larger noncap region (29). The ExsY/BxpB scaffold appears to be restricted primarily to the noncap region of the wild-type exosporium. The cap appears to employ an analogous scaffold formed by paralogs of ExsY and BxpB, namely, CotY and ExsFB, respectively (16, 26, 30). ExsFB is required for the attachment of the small amount of BclA that does not involve BxpB (17, 18).

The crystal structure of BxpB also suggests an orientation for BxpB trimers within the basal layer and a mechanism for BxpB attachment to ExsY. Although the first 19 residues of BxpB are presumably disordered and thus not visible in the crystal structure, the position of residue Thr20 suggests that these disordered residues are most likely positioned at the bottom of the structure shown in Fig. 1B. These residues are particularly noteworthy because they include the longest stretch of nonidentical amino acids when the sequences of BxpB and ExsFB are compared. These 167-residue proteins exhibit 78% sequence identity, but only six of the first 18 residues (and three between positions 5 and 18) are identical. It seems reasonable to suspect that the amino-terminal residues of BxpB and ExsFB (i.e., residues 1–18) are responsible, at least in part, for the differential localization of the two proteins within the exosporium: BxpB binding to ExsY in the noncap region and ExsFB binding to CotY in the cap. Furthermore, the only cysteine residues in BxpB are located within the amino-terminal region at positions 6 and 13. Twelve of the 152 amino acids of ExsY are cysteine residues, and disulfide bond formation involving at least some of these cysteines is important for stabilizing the self-assembled ExsY scaffold (26). Perhaps, other ExsY cysteines could participate in disulfide bond formation with one or both cysteines of BxpB, allowing covalent attachment of the two proteins. A similar situation could occur with ExsFB, which contains one cysteine residue at position 13, and CotY, in which 14 of its 156 residues are cysteines. Taken together, the observations above suggest that the bottom of the BxpB trimer structure shown in Fig. 1B directly contacts an underlying ExsY scaffold and that this contact could be stabilized by disulfide bond formation between BxpB and ExsY.

According to the proposed orientation for BxpB trimers in the basal layer, the top of the structure shown in Fig. 1B would contact the amino-terminal region of an attached BclA trimer. This interaction could be between individual monomers of BclA and BxpB or something more complex. In either event, the region of BxpB proposed to interface with the BclA NTD is comprised of three loops, two of which are involved in binding Ca^2+^ (Fig. 1A and B). The importance of Ca^2+^ in BxpB structure and function is unknown, but Ca^2+^ is abundant in spores (31).

Although the crystal structure of the BxpB trimer resembles that of a trimer of the CTD of BclA in several ways, BxpB trimers are much less stable than BclA CTD trimers when treated with heat and detergent. On the other hand, when BxpB trimers are incubated with a peptide containing residues 20–38 of the NTD of BclA, they form a complex with an apparent molecular mass of 55–65 kDa that is as stable to heat and detergent as either BclA CTD trimers or BxpB-BclA complexes extracted from spores. This result provides, for the first time, unambiguous evidence for a direct and highly stable interaction between BxpB and the NTD of BclA. In addition, upon analysis by SDS-PAGE, it appears that 52 kDa BxpB trimers dissociate from the putative BxpB trimer-BclA NTD peptide complexes and that these trimers are much more stable than those formed by purified BxpB. Apparently, binding of the BclA NTD peptide to a BxpB trimer induces a change in the structure of the trimer to a more stable form capable of entrapping the NTD of BclA, and this stable structure persists even after dissociation of the peptide. The structure of BxpB within the BxpB trimer-BclA NTD peptide complex is presently being investigated.

We were also able to recapitulate the formation of BxpB trimer-BclA NTD peptide complexes *in vivo* using a *B. anthracis* variant (CLT406) that produces the NTD of BclA instead of the full-length protein. These complexes were similar in size and exhibited similar stabilities to those formed *in vitro*. The heterogeneity in the complex bands, formed both *in vitro* and *in vivo*, indicate variability in the stoichiometry of complex components (e.g., number of peptides) and/or the shape of the complexes. We also observed high levels of 52- kDa trimeric BxpB with strain CLT406, which was not observed with wild-type and *ΔbclA* strains. These results indicate that the interactions between BxpB and the BclA NTD and NTD-induced changes in BxpB trimer structure are the same with purified proteins in a test tube as they are during spore development.

The extreme stability of BxpB-BclA complexes extracted from *B. anthracis* spores and the virtual absence of free BclA in this extract raised the possibility that the connection between the two proteins was covalent (17). It was also speculated that such a covalent connection was linked to the proteolytic cleavage of the BclA NTD between residues 19 and 20 (17). However, the formation of BxpB-BclA complexes and their insertion into the basal layer were subsequently shown to occur prior to cleavage of the BclA NTD (20). Furthermore, we have searched for evidence of a covalent linkage between BxpB and BclA by proteolytically digesting BxpB-BclA complexes extracted from spores and exhaustively analyzing the resulting peptides by LC-MS/MS. No cross-linked peptides containing fragments of both BxpB and BclA were detected. Thus, the case for a covalent linkage between BxpB and BclA is weak.

On the other hand, the results presented in this paper make a strong argument for a highly stable but noncovalent interaction between BxpB and BclA. The structural change in BxpB trimers induced by the BclA NTD, which results in a highly stable trimer, could also entrap the BclA NTD in a similarly stable complex. Such entrapment could involve the multiple loops at the top of BxpB as well as other exposed regions of the protein. In addition, BxpB trimers appear to dissociate from BxpB trimer-BclA NTD peptide complexes, indicating that their association with the BclA NTD is noncovalent. In contrast, BclA does not readily dissociate from BxpB-BclA complexes. This difference suggests that trimerization of BclA enhances the stability of BxpB-BclA complexes, an effect that appears to be independent of BclA glycosylation (14). Furthermore, stable BxpB trimer-BclA NTD peptide complexes form *in vitro* in the absence of other protein factors and an obvious source of energy that would be required for covalent attachment. The involvement of proteolytic cleavage of the BclA NTD in this process is precluded by the use of a BclA NTD peptide containing only residues 20–38. If the interaction between BxpB and BclA is indeed noncovalent, elucidating the exact nature of the linkage will require additional structural analysis of the BxpB-BclA complex. A detailed understanding of BxpB-BclA attachment will be generally useful as many spore-forming bacteria, including important pathogens, appear to use the same or analogous mechanisms to attach BclA and BclA-like filamentous trimers to the basal layer of the exosporium (26, 32).

Finally, it is necessary to discuss a recent publication by Durand-Heredia et al. (33) that suggests a fundamentally different role for BxpB in exosporium assembly than that described in this paper. The critical difference can be summarized by a single statement in Durand-Heredia et al.: “BclA may be stably attached to an exosporium basal layer protein, but that protein is not BxpB.” Essentially, two arguments led to this conclusion, and each will be discussed separately.

First, according to Durand-Heredia et al., the component of the BxpB-BclA complex that directs its insertion into the basal layer is BclA, not BxpB. In fact, in the absence of BclA, incorporation of BxpB into the basal layer is very inefficient, a co-dependence model first proposed in 2011 (34). There is clear evidence that contradicts the co-dependence model. This model was first refuted in 2014 in a paper that included the cryo-electron microscopic analysis of exosporia of wild-type, *ΔbclA*, and *ΔbxpB* spores of *B. anthracis* mentioned in the preceding text. The relevant point here is that the structures of wild-type and Δ*bclA* exosporia are indistinguishable, except for the presence of BclA-containing filaments in the wild-type exosporium. In both cases, the exosporium was highly ordered, and the basal layer exhibited the same level of putative BxpB-containing projections to which filaments are attached in the wild-type exosporium. In sharp contrast, the absence of BxpB resulted in a disordered basal layer that lacked projections, a readily detectable phenotype. Accordingly, the absence of this phenotype in the *ΔbclA* exosporium indicates a full complement of BxpB without the aid of BclA. Additionally, previous studies have shown that comparable levels of BxpB-containing material are extracted from wild-type and *ΔbclA* spores (17). This same result can be seen in Fig. 6 of this paper. Taken together, these data indicate that BxpB does not require BclA for efficient (wild-type level) incorporation into the basal layer.

Second, Durand-Heredia et al. state that the actual tight-binding partner for BclA is an unidentified basal layer protein with a mass of 28–32 kDa, hereafter called P30. The most definitive evidence offered is that, *in vivo*, a His_12_-tagged version of the BclA NTD tightly associates with P30, a complex detected with antiserum against the His tag. This experiment is essentially the same as that with strain CLT406 shown in Fig. 6, where abundant BxpB trimer-BclA NTD peptide complexes (and BxpB trimers) were detected. However, the BclA NTD-containing complexes in the two experiments appear to be different with apparent molecular masses of 30–40 kDa and 55–65 kDa. It does not appear that Durand-Heredia et al. probed the 30 to 40-kDa complex with anti-BxpB antibodies to exclude the possibility that the complex contained BxpB. At this point, an assessment of the role of P30 in BclA attachment must await its identification. It is possible that the BclA NTD stably interacts with P30 after the insertion of BxpB-BclA complexes into the basal layer, perhaps associated with BclA NTD cleavage. However, extensive published genetic, biochemical, and structural data and the data presented in our paper make an extremely strong case that BxpB, and to a lesser extent ExsFB, are the only basal layer proteins involved in the primary attachment of BclA to the basal layer.

## MATERIALS AND METHODS

### Bacterial strains

The Sterne 34F2 avirulent veterinary vaccine strain of *B. anthracis* was used as the wild-type strain and as the parent in strain constructions. Two mutant variants of the Sterne strain were constructed by an allelic exchange on the chromosome essentially as previously described (14, 17). These constructs were cured of intermediate plasmids vehicles used to introduce changes into the *bclA* locus. For strain CLT306 (*ΔbclA*), the entire *bclA* gene was deleted and replaced with a spectinomycin resistance cassette. For strain CLT406, a TAA stop codon was inserted into the *bclA* gene immediately after codon 38. This construction also included a spectinomycin resistance cassette inserted after the *bclA* transcription terminator. All constructions were confirmed by PCR amplification and DNA sequencing of the relevant regions of the chromosome. Strain CLT307 (*ΔbxpB*), another Sterne variant in which codons 1–163 of the *bxpB* gene were replaced with a spectinomycin resistance cassette, was described previously (17).

### Preparation of BxpB and the BclA CTD

BxpB was expressed and purified essentially as previously described (17). Briefly, the *bxpB* gene of the Sterne strain was expressed in *E. coli* strain RY3041 (BL21(DE3) *slyD*::Tn10) from a modified version of expression vector pET15B (pCLT1733) producing a recombinant BxpB with a His_6_-tag and a Factor Xa cleavage site immediately preceding the BxpB initiating methionine. Recombinant BxpB was purified under native (and reducing) conditions by immobilized metal affinity chromatography (QIAGEN or Cube Biotech), dialyzed into Xa buffer, and cleaved by Factor Xa (New England BioLabs). Passage through a second affinity column removed the His-tag-containing amino-terminal polypeptide, and Factor Xa was removed using a Factor Xa capture kit (Novagen). Purified BxpB was concentrated using an Amicon Ultra-4 centrifugal filter. A His_6_-tagged version of the BclA CTD was expressed in pCLT1218/RY3041 and purified by immobilized metal affinity chromatography essentially as previously described (12). The resulting recombinant protein, which was dialyzed into Xa buffer, contains the amino-terminal sequence MGSSHHHHHHSSGLVPRGSHNIEGR fused to the 134 amino acids of the CTD of BclA. The concentrations of purified BxpB and His_6_-tagged BclA CTD were measured spectroscopically at 280 nM using calculated molar extinction coefficients (ExPASy). All genetic constructions encoding proteins described above were confirmed by DNA sequence analysis.

### BclA NTD peptide

A ≥98% pure preparation of a peptide containing BclA residues 20–38 was purchased from GenScript. The sequence was confirmed by mass spectrometry.

### Crystallization, X-ray diffraction data collection, and structure determination of BxpB

Purified BxpB was concentrated by ultrafiltration to a final concentration of 28 mg/mL (1.6 mM) and crystallized using the sitting drop vapor diffusion technique. The reservoir solution containing 30% Jeffamine M-600, pH 7.0, and protein solution were mixed in a 1:1 ratio and equilibrated at 22°C. For X-ray data collection, a single crystal was frozen in liquid nitrogen without cryo-preservation. Diffraction data extending to 1.4 Å were collected at APS SER-CAT ID synchrotron beam line.22ID on Dectris Eiger X 16M detector at 100K. The crystal structure was solved by a molecular replacement method using Phaser (35) and a homology model generated using RoseTTAFold (36). Phenix version 1.19.2 (37) and Coot (38) were used for refinement and model building. The crystal structure has been refined to 1.4 Å resolution. The final R and R free values are 0.1763 and 0.1894. Details of data collection and refinement statistics are listed in Table S1.

### SEC-MALS

A 100-µL sample of BxpB was injected onto a WTC-MP015N5 column, 4.6 by 300 mm I.D; particle size, 5 mm; pore size, 150 Å (Wyatt Technology, Santa Barbara, CA) on a Shimadzu Prominence HPLC System (Shimadzu Corp., Kyoto, Japan) with an isocratic run at 0.3 mL/min for 17 min, and a solution of 20 mM Tris-HCl (pH 8.0), 100 mM NaCl, 2 mM CaCl2, and 2 mM TCEP was used as the mobile phase. UV absorbance was set at 280 nm using Shimadzu HPLC Explorer software (Shimadzu Corp., Kyoto, Japan). A DAWN 8 MALS detector (Wyatt Technology, Santa Barbara, CA), set at 659 nm, and an Optilab refractometer (Wyatt Technology, Santa Barbara, CA) were used in tandem for detection. Bovine serum albumin (Wyatt Technology, Santa Barbara, CA) was used to normalize the static light scattering detector. The delay volume, band broadening parameters, and the light scattering and differential refractive index measurements were analyzed using Astra 8 software (Wyatt Technology, Santa Barbara, CA).

### Gel electrophoresis and immunoblotting

Purified and exosporium proteins in sample buffer containing 62.5 mM Tris-HCl (pH 6.8), 2% SDS, 100 mM DTT, 0.012% bromophenol blue, and 10% (v/v) glycerol were separated by SDS-PAGE in a NuPAGE 4–12% Bis-Tris gel (Invitrogen) and visualized by staining with Coomassie brilliant blue. Before loading samples of extracted exosporium proteins, insoluble material was removed by centrifugation for 1 min at 18,000 × *g*. For immunoblotting, proteins were electrophoretically transferred from an unstained polyacrylamide gel to a nitrocellulose membrane and treated as described in the manual for the Bio-Rad Immun-Blot HRP Assay kit. The primary antibody used for immunoblotting was the mouse anti-BxpB mAb 10-44-1, prepared as previously described (6, 19).

### Preparation of spores

Spores were prepared by growing *B. anthracis* strains at 37°C on LB agar plates until sporulation was complete, typically 3 d. Spores were washed from plates with cold (4°C) sterile water, collected by centrifugation, washed twice with cold water, and stored in water at 4°C for 12–24 h. Spores were then purified by sedimentation through a two-step gradient of 20% and 45% Isovue-300 (Bracco Diagnostics) and washed three times with cold water. Spores were stored at −20°C and quantitated microscopically with a Petroff-Hausser counting chamber.

### Mass spectrometry

For protein analysis by mass spectrometry, a Coomassie stained protein band was excised from a polyacrylamide gel and digested with sequencing grade chymotrypsin (Promega, Madison, WI) (39). Proteolytic fragments were analyzed by LC-MS/MS with electrospray ionization using a Thermofisher Hypersil Gold 80 Å reverse-phase column (Torrance, CA) and Exion UHPLC linked to a SCIEX 5600 Triple-Tof mass spectrometer (SCIEX, Toronto, Canada). The MS/MS data were processed to provide protein identifications using an in-house *Protein Pilot 5.0* search engine (Sciex, Toronto, Canada) using the *B. anthracis* UniProt protein database and a chymotrypsin plus missed cleavage digestion parameter. Sequences identified in the software were verified by manual *de novo* sequencing for authenticity against the known sequences of BclA and/or BxpB proteins.

## Data Availability

Atomic coordinates and structure factors for BxpB have been deposited in the Protein Data Bank: PDB ID: 3D02 and Deposition ID D_1000263664.

## SUPPLEMENTAL MATERIAL

Fig. S1 mbio.01172-23-s0001.tif Topology diagram of BxpB generated by using PDBsum.

Fig. S2 mbio.01172-23-s0002.tiff Ca^2+^ coordination within the BxpB crystal structure.

Fig. S3 mbio.01172-23-s0003.tif SEC-MALS analysis of BxpB in PBS.

Fig. S4 mbio.01172-23-s0004.tif Examining the cross-reactivity of anti-BxpB mAb 10-44-1.

Table S1 mbio.01172-23-s0005.docx X-ray data collection and refinement statistics.

ASM does not own the copyrights to Supplemental Material that may be linked to, or accessed through, an article. The authors have granted ASM a non-exclusive, world-wide license to publish the Supplemental Material files. Please contact the corresponding author directly for reuse.
