# Supplementary figures and images for: Crystal structure and induced stability of trimeric BxpB: implications for the assembly of BxpB-BclA complexes in the exosporium of Bacillus anthracis
Source: mBio. 2023 Jun 29;14(4):e01172-23. doi: 10.1128/mbio.01172-23 (PMC10470788; doi:10.1128/mbio.01172-23)

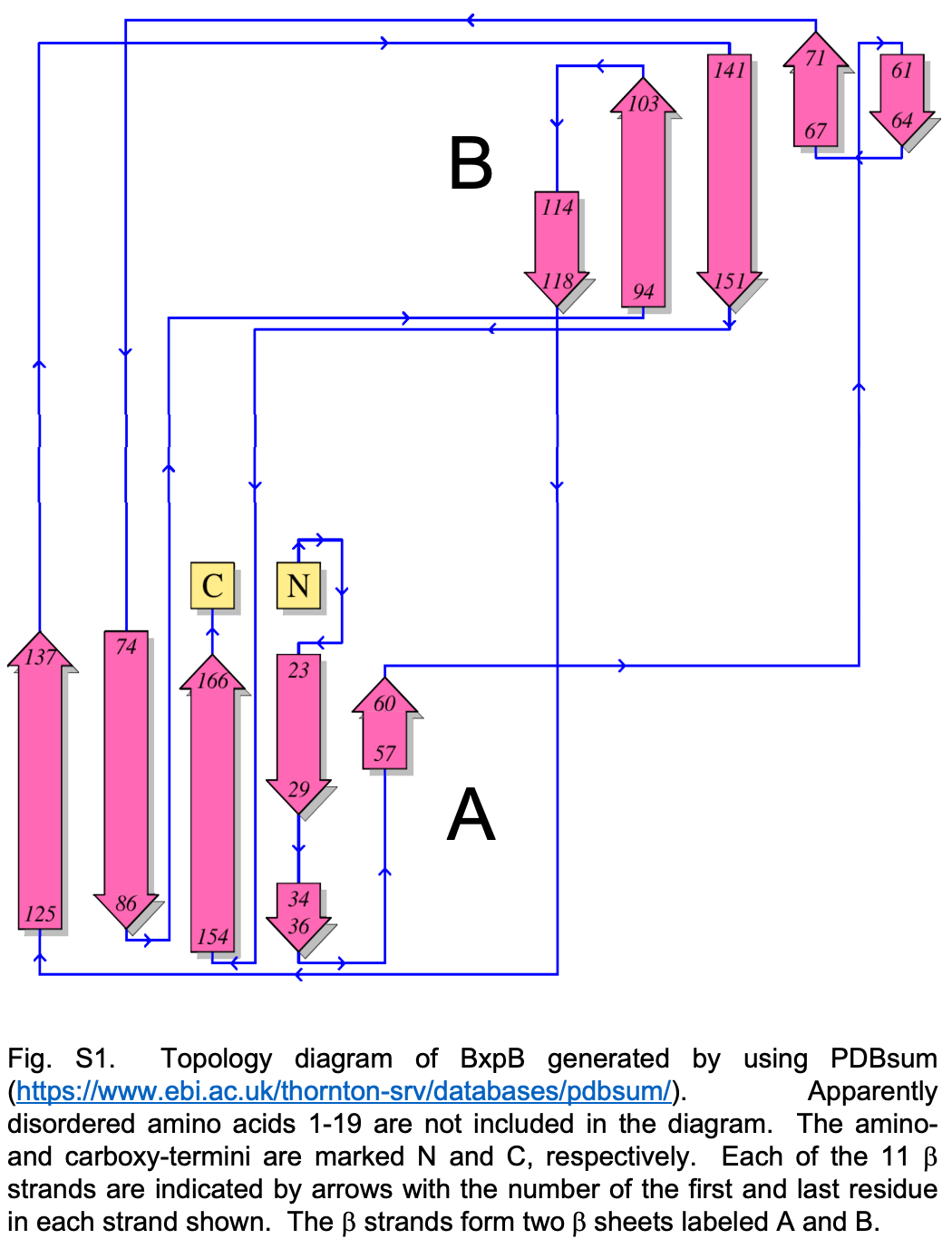

Supplement: Fig. S1 — Topology diagram of BxpB generated by using PDBsum. [file mbio.01172-23-s0001.tif]

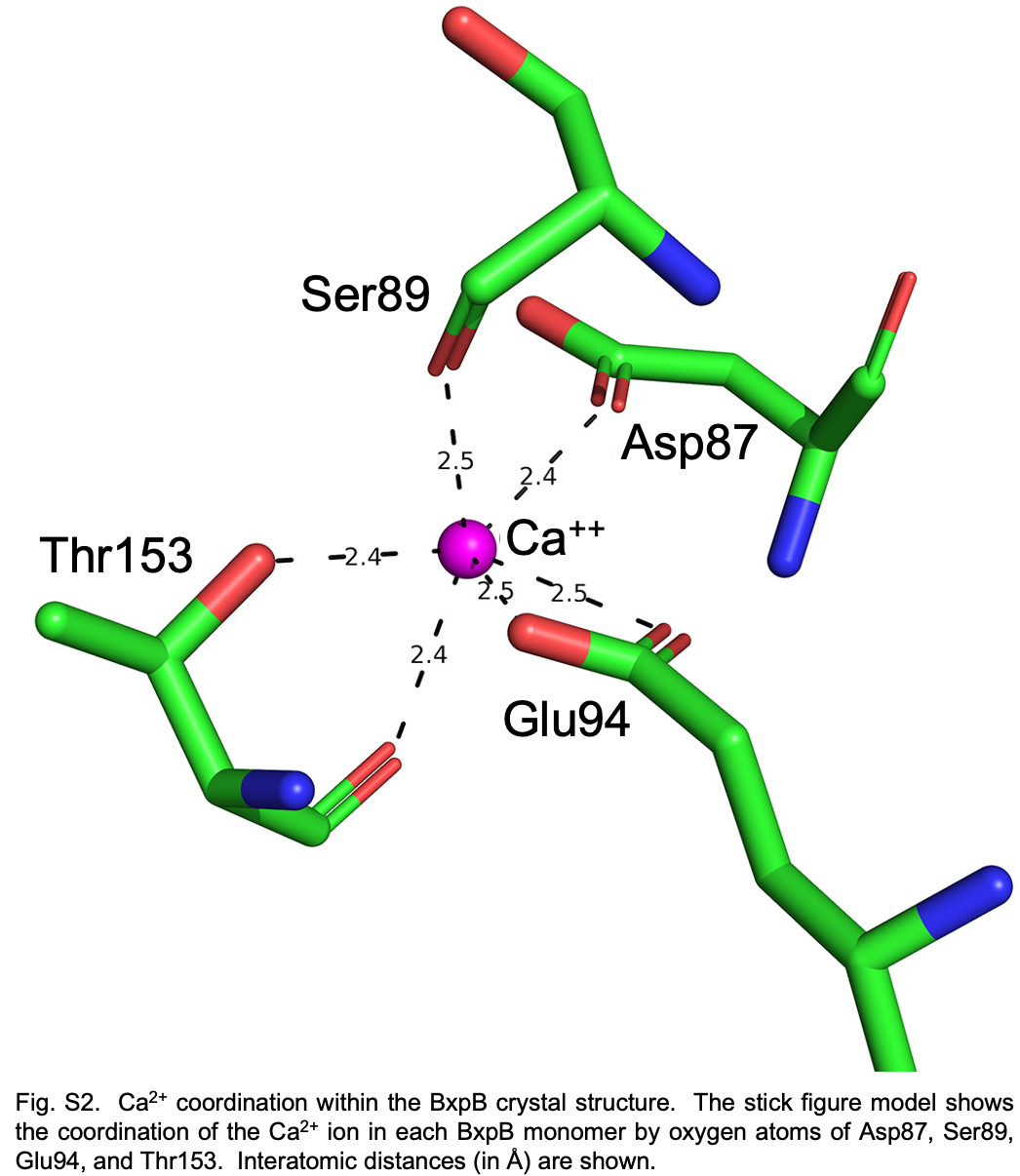

Supplement: Fig. S2 — Ca2+ coordination within the BxpB crystal structure. [file mbio.01172-23-s0002.tiff]

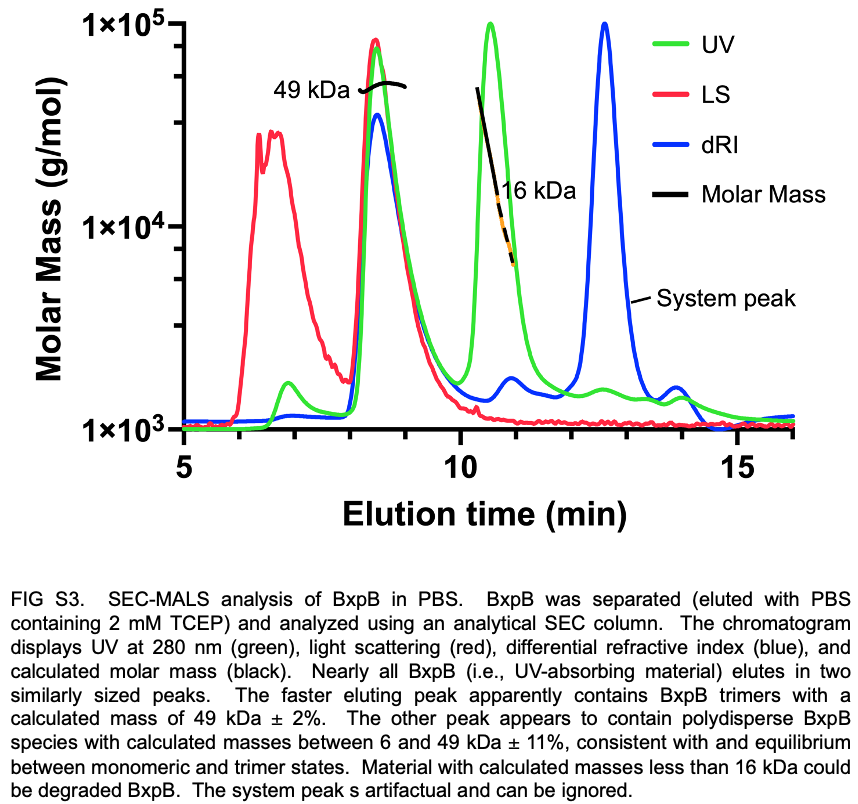

Supplement: Fig. S3 — SEC-MALS analysis of BxpB in PBS. [file mbio.01172-23-s0003.tif]

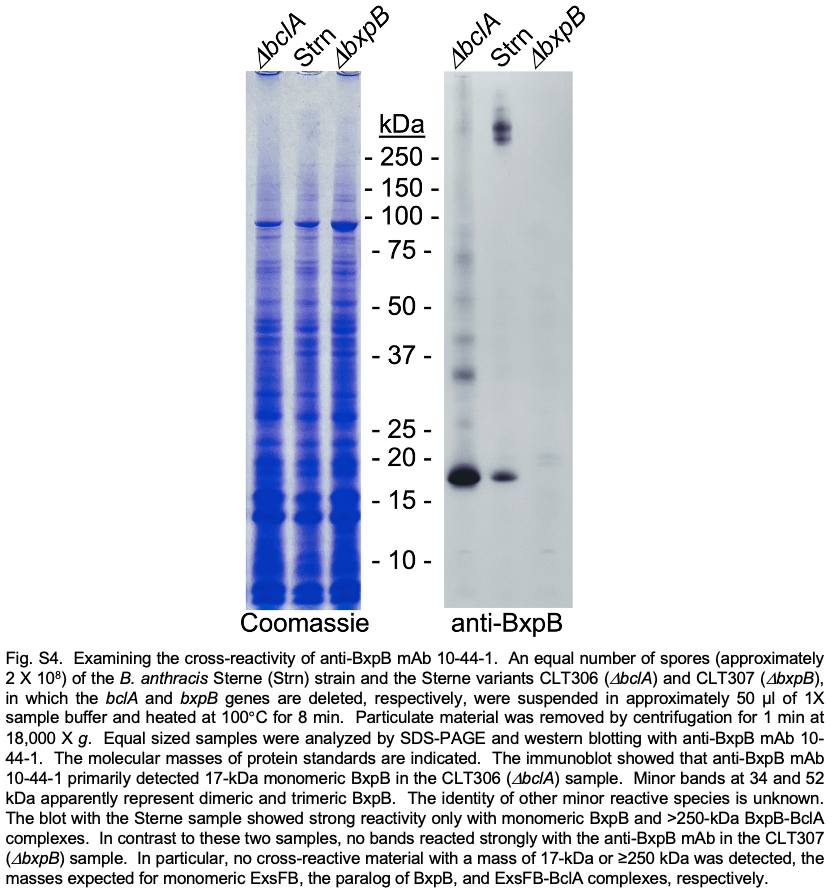

Supplement: Fig. S4 — Examining the cross-reactivity of anti-BxpB mAb 10-44-1. [file mbio.01172-23-s0004.tif]
