# Supplementary material for: Crystal structure and induced stability of trimeric BxpB: implications for the assembly of BxpB-BclA complexes in the exosporium of Bacillus anthracis
Source: mBio. 2023 Jun 29;14(4):e01172-23. doi: 10.1128/mbio.01172-23 (PMC10470788; doi:10.1128/mbio.01172-23)
Supplement: Table S1 — X-ray data collection and refinement statistics. [file mbio.01172-23-s0005.docx]

Table S1. X-ray data collection and refinement statistics

Wavelength (Å) 1.0

Temperature (K) 100

Space group I23

Cell parameters a = b = c = 99.90

α = β = γ = 90

Resolution range 70.64-1.40 (1.42-1.40)*

No. of unique reflections 32721 (1581)

Completeness (%) 100 (100)

Multiplicity 30.8 (16.5)

[I/σ(I)] 26.0 (2.83)

CC_1/2_ 1.00 (0.854)

Rmerge 0.08 (0.96)

R_pim_  0.014 (0.239)

Overall B factor from Wilson plot (Å^2^) 17.05

**Refinement statistics**

Resolution range 49.95-1.40 (1.44-1.40)

Completeness (%) 99.99 (100)

No. of reflections 32719 (2799)

Reflections in the test set 1582 (114)

R work (%) 17.49 (21.67)

R free (%) 18.60 (23.87)

No. of non-H atoms 1180

No. of protein residues 148

R. m. s deviations

Bond length (Å) 0.012

Bond angles (°) 1.28

Mean overall B factors (Å^2^) 18.33

Ramachandran plot

Most favored (%) 97.95

Allowed (%) 2.05

Outliers (%) 0.00

PDB entry D02

*Figures in parentheses represent highest resolution shell.
